# Sodium Butyrate Protects against Severe Burn-Induced Remote Acute Lung Injury in Rats

**DOI:** 10.1371/journal.pone.0068786

**Published:** 2013-07-11

**Authors:** Xun Liang, Ren-Su Wang, Fei Wang, Sheng Liu, Feng Guo, Li Sun, Yong-Jie Wang, Ye-Xiang Sun, Xu-Lin Chen

**Affiliations:** Department of Burns, the First Affiliated Hospital of Anhui Medical University, Hefei, Anhui, PR China; The Ohio State University, United States of America

## Abstract

High-mobility group box 1 protein (HMGB1), a ubiquitous nuclear protein, drives proinflammatory responses when released extracellularly. It plays a key role as a distal mediator in the development of acute lung injury (ALI). Sodium butyrate, an inhibitor of histone deacetylase, has been demonstrated to inhibit HMGB1 expression. This study investigates the effect of sodium butyrate on burn-induced lung injury. Sprague–Dawley rats were divided into three groups: 1) sham group, sham burn treatment; 2) burn group, third-degree burns over 30% total body surface area (TBSA) with lactated Ringer’s solution for resuscitation; 3) burn plus sodium butyrate group, third-degree burns over 30% TBSA with lactated Ringer’s solution containing sodium butyrate for resuscitation. The burned animals were sacrificed at 12, 24, and 48 h after burn injury. Lung injury was assessed in terms of histologic changes and wet weight to dry weight (W/D) ratio. Tumor necrosis factor (TNF)-α and interleukin (IL)-8 protein concentrations in bronchoalveolar lavage fluid (BALF) and serum were measured by enzyme-linked immunosorbent assay, and HMGB1 expression in the lung was determined by Western blot analysis. Pulmonary myeloperoxidase (MPO) activity and malondialdehyde (MDA) concentration were measured to reflect neutrophil infiltration and oxidative stress in the lung, respectively. As a result, sodium butyrate significantly inhibited the HMGB1 expressions in the lungs, reduced the lung W/D ratio, and improved the pulmonary histologic changes induced by burn trauma. Furthermore, sodium butyrate administration decreased the TNF-α and IL-8 concentrations in BALF and serum, suppressed MPO activity, and reduced the MDA content in the lungs after severe burn. These results suggest that sodium butyrate attenuates inflammatory responses, neutrophil infiltration, and oxidative stress in the lungs, and protects against remote ALI induced by severe burn, which is associated with inhibiting HMGB1 expression.

## Introduction

Pulmonary pathology in major thermal injury is found in 30% to 80% of burn fatalities [1]. Acute lung injury (ALI) is a leading complication in patients with extensive burns in which the burned area exceeds 30% of the total body surface area (TBSA) [2]. ALI and its extreme manifestation, acute respiratory distress syndrome (ARDS), are the well-documented major cause of morbidity and mortality in burned patients admitted to the hospital, especially in patients with combined smoke inhalation injury or delayed resuscitation [2]–[4]. Although the pathophysiologic mechanisms underlying burn-induced ALI remain incompletely elucidated, growing evidence from experimental and clinical studies shows that systemic inflammatory response and oxidative stress play a central role in the development of ALI [5]–[7].

High mobility group box protein 1 (HMGB1), known as an abundant, non-histone architectural chromosomal protein, is highly conserved across different species [8]. It was originally discovered as a DNA binding protein that facilitates DNA replication and repair [9]–[11]. Presently, HMGB1 participation in innate and specific immune responses has been revealed. HMGB1 acts as an alarmin and is responsible for the production of proinflammatory cytokines, contributes to the pathogenesis of diverse inflammatory and infectious disorders when passively released into the extracellular environment from necrotic cells or actively produced by various cell types upon cellular stress/damage [11], [12].

Meanwhile, HMGB1 has been identified as a distal mediator of acute inflammatory lung injury [13], [14]. HMGB1 concentrations are increased in the plasma and lung epithelial lining fluid of patients with ALI [15]. Moreover, HMGB1 expression in blood and bronchoalveolar lavage fluid (BALF) is correlated with poor outcomes in lung injury patients [16]. In endotoxin-induced ALI, administration of anti-HMGB1 antibodies before or after endotoxin exposure decreases the migration of neutrophils into the lungs as well as lung edema [17]. Recent studies show that the HMGB1 A box, a specific blocker of endogenous HMGB1, attenuates neutrophil infiltration, decreases the expression of chemokines and proinflammatory cytokines, and prevents ALI [18], [19]. These results suggested that HMGB1 has potent inflammatory properties that contribute to the development of ALI.

Sodium butyrate, an inhibitor of histone deacetylase, has been reported that it could provide an anti-inflammatory effect and could inhibit HMGB1 expression in sepsis [20], ischemic stroke [21], myocardial ischemia/reperfusion [22], and lipopolysaccharide (LPS)-induced ALI [23]. Thus, we hypothesized that sodium butyrate may protect against severe burn-induced remote ALI by inhibiting HMGB1 expression. In the present study, the major purpose was to investigate whether treatment of sodium butyrate protects against burn-induced lung injury as well as the inflammatory response and oxidative stress in severely burned rats.

## Materials and Methods

### Animals

Healthy adult female Sprague–Dawley rats weighing 200 g to 250 g were used throughout the study. All experimental manipulations were undertaken in accordance with the Guide for the Care and Use of Laboratory Animals by the National Institutes of Health, with the approval of the animal experimental ethics committee of Anhui Medical University, China. Animals were fed a standard animal diet with food and tap water *ad libitum* and acclimatized to their environment for at least 1 week prior to the experiment.

### Burn Procedure

The rats were anesthetized with pentobarbital (30 mg/kg) intraperitoneally, shaved on the dorsal and lateral surfaces, and secured on a constructed template device. The surface area of the skin exposed through the template device was immersed in 98°C water for 12 s on the dorsal surface. All were quickly dried after each exposure to avoid additional injury. A 30% TBSA full-thickness dermal burn was then obtained using this technique.

### Experimental Design

**Figure 1:**
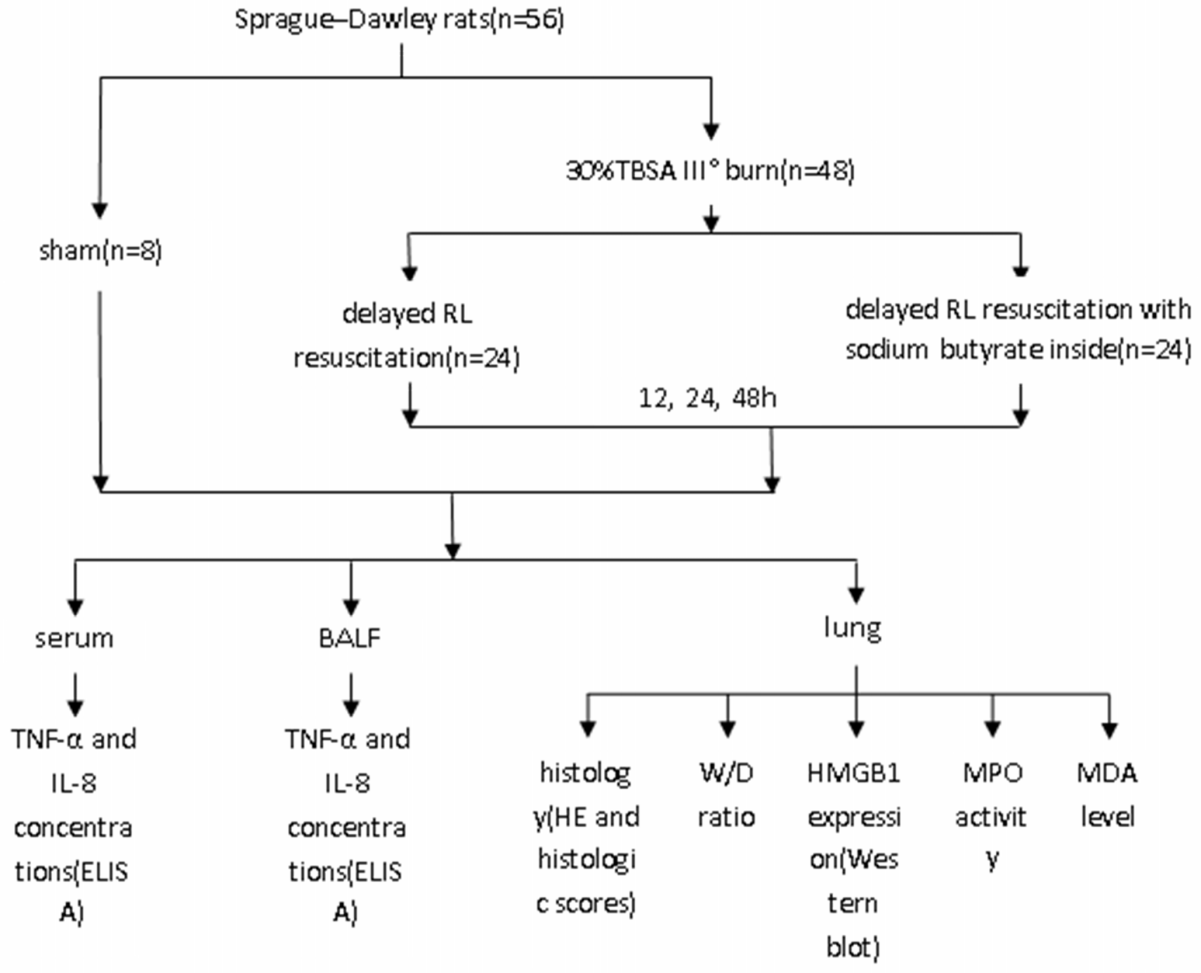
Experiment design.

A total of 48 rats were subjected to 30% TBSA full-thickness burns and randomized into a burn group (n = 24) and a burn plus sodium butyrate group (n = 24). The burn control rats were then resuscitated with an intraperitoneal injection of 2 ml/kg/TBSA of lactated Ringer’s solution at 6, 12, and 36 h post burn. Sodium butyrate was diluted 1∶150 in lactated Ringer’s solution. The rats in burn plus sodium butyrate group were treated with sodium butyrate lactated Ringer’s solution in the same manner. That is, the amount of sodium butyrate given was 400 mg/kg. Subsequently, the burned animals were sacrificed at 12, 24, and 48 h after injury for bronchoalveolar lavage and tissue sampling. A separate group of sham burned rats (n = 8) was subjected to an identical preparation except for immersion in room temperature water and fluid resuscitation. The experimental design was show in Figure 1.

### Measurement of Tumor Necrosis Factor (TNF)-α and Interleukin (IL)-8 Concentrations in BALF and Serum

The TNF-α and IL-8 concentrations were measured in the supernatant fluids of bronchoalveolar lavage samples and serum by a “sandwich” enzyme-linked immunosorbent assay (ELISA) using TNF-α and IL-8 ELISA kits for rats (Shanghai Biovol Technologies Co., Shanghai, China) according to the manufacturer’s instructions.

### Histological Examination

The lung specimens were fixed in 10% formalin. The tissues were dehydrated, embedded in paraffin, cut into 5 µm sections, and mounted. The tissues were then stained with hematoxylin and eosin after deparaffinization. Histologic changes were graded by two blinded examiners. The lung tissues were graded for intra-alveolar edema, intra-alveolar hemorrhage, and neutrophil infiltration from 0 to 4 (0, absent; 1, mild; 2, moderate; 3, severe; 4, overwhelming) for a maximum score of 12, as described previously by Gloor et al. [24].

### Measurement of Lung Wet Weight to Dry Weight (W/D) Ratio

To quantify the magnitude of pulmonary edema, we examined the W/D ratio of the lungs. The left upper lung was excised after the rat was sacrificed. The exudate and blood on the surface were blotted off with filter paper, and the lung was weighed to obtain the “wet” weight. Afterwards, the lung was dried in an oven at 75°C for 72 h to obtain the “dry” weight. The W/D ratio was then calculated.

### Western Blot Analysis of Pulmonary HMGB1 Expression

The HMGB1 expression in the lung tissues was determined by western blot analysis. All specimens had a wet weight of 100 mg. The extraction buffer contained 20 mM Tris (pH 7.5), 150 mM NaCl, 1 mM EDTA, 1 mM EGTA, 1% Triton X-100, 2.5 mM sodium pyrophosphate, 1 mM β-glycerophosphate, 1 mM Na_3_VO_4_, and 1 µg/mL leupeptin. Phenylmethylsulfonyl fluoride (1 mM) was added immediately before use. The samples were homogenized in 4 volumes of extraction buffer on ice. All debris was removed by centrifugation at 900 ×*g* at 4°C for 10 min. The supernatant obtained liquid was used as the whole cell lysate. The protein concentrations were determined with bicinchoninic acid protein assay reagent (Pierce, Rockford, IL).

Protein extracts were separated by sodium dodecyl sulfate-polyacrylamide gel electrophoresis and electrotransferred onto a nitrocellulose membrane. Then, the blots were blocked with 5% nonfat dry milk and Tris-buffered saline with 0.1% Tween-20. The membrane was then incubated overnight at 4°C with primary polyclonal rabbit antibodies specific for HMGB1 (Cell Signaling Technology, Beverly, MA) at a dilution of 1∶1000 followed by horseradish peroxidase–conjugated anti-rabbit secondary antibody (Santa Cruz Biotechnology) at a dilution of 1∶2000. After three washings, the immunocomplexes were detected using an electrochemiluminescence plus detection kit (Amersham, Little Chalfont, UK) and were exposed to film.

### Detection of Myeloperoxidase Activity in Lung Tissue

As an index for neutrophil sequestration in lung tissue, the activity of myeloperoxidase (MPO) was measured with an MPO assay kit (Nanjing Jiancheng Bioengineering Institute, Nanjing, China) according to the manufacturer’s instructions. Briefly, frozen lung tissues were thawed and homogenized (1∶10, w/v) in normal saline. The homogenate was diluted (1∶1) with solution B, and then the samples were assayed spectrophotometrically for MPO activity following the recommendations of the manufacturer. MPO catalyzes the redox reaction of peroxide and 3,3′,5,5′-tetramethylbenzidine and produces yellow compounds [25]. The absorbance at 460 nm was determined using a 721 spectrophotometer (Shanghai, China). One unit of MPO is defined as the amount of MPO capable of degrading one micromole of peroxide per gram of wet lung tissue at 37°C. The results are expressed as MPO units per gram of wet lung tissue.

### Measurement of Lung Malondialdehyde (MDA) Concentration

The lung concentrations of MDA, an indicator of lipid peroxidation and oxidative stress [26], was measured via the thiobarbituric acid colorimetric method using a commercial kit (Nanjing Jiancheng Bioengineering Institute, Nanjing, China) according to the manufacturer’s instructions. Briefly, frozen lung tissues were weighed and homogenized (1∶10, w/v) in normal saline in an ice bath. The homogenates were centrifuged at 900 ×*g* for 10 min at 4°C. The MDA concentrations in the supernates were measured strictly following the recommendations of the manufacturer. Absorbance at 532 nm was measured using a spectrophotometer. The protein concentrations in the supernates were determined using the Bradford method, and the MDA concentration is expressed as nanomoles per milligram of protein.

### Statistical Analysis

All data are expressed as mean ± SEM. Statistical comparisons among groups were analyzed using a one-way ANOVA and Student–Newman–Keuls q test. Differences with p<0.05 were considered statistically significant.

## Results

### Sodium Butyrate Decreases the Concentrations of Local and Systemic Inflammatory Mediators Induced by Severe Burn Injury

**Figure 2:**
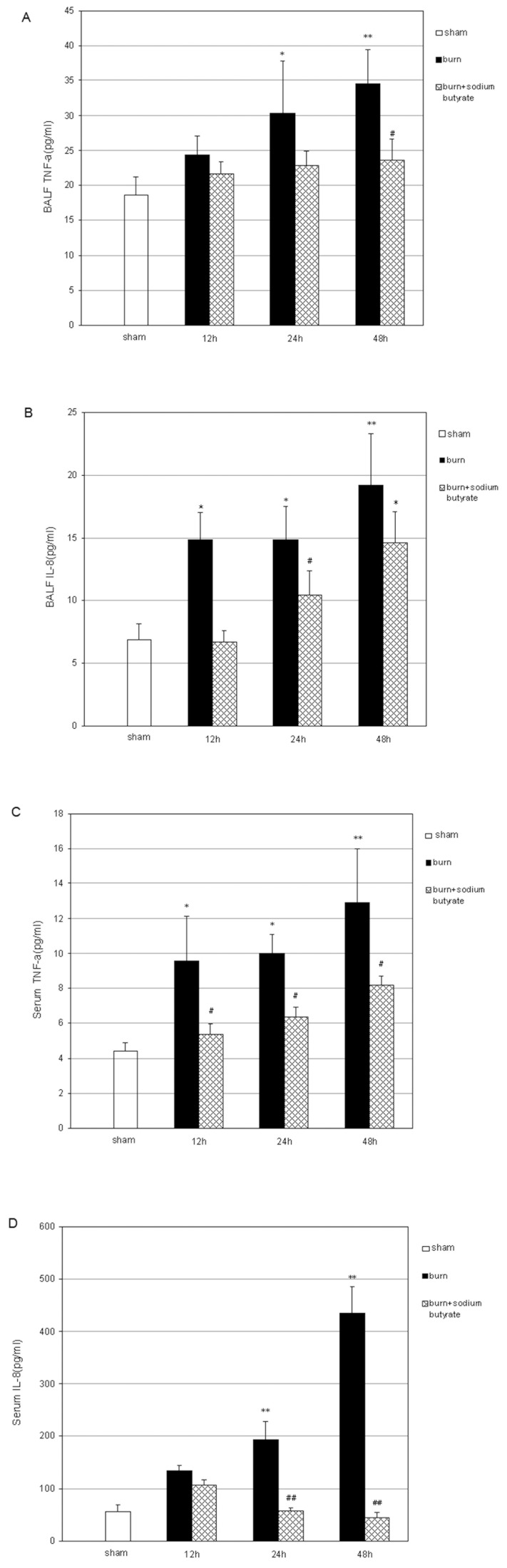
Effect of sodium butyrate on the concentrations of inflammatory mediators in BALF and serum after burn injury. After the 30% TBSA full-thickness burn injury, the TNF-α and IL-8 concentrations were markedly increased in BALF and serum at 12 h post burn and remained in high values up to 48 h. Sodium butyrate administration significantly suppressed the burn-induced increases in TNF-α and IL-8 concentrations in BALF and serum at 24 and 48 h after injury. ELISA measured the (A) BALF TNF-α concentrations, (B) BALF IL-8 concentrations, (C) serum TNF-α concentrations, and (D) serum IL-8 concentrations. Results are given as mean ± SEM (n = 8). *p<0.05, **p<0.01, vs. sham, ANOVA; ^#^p<0.05, ^##^p<0.01, vs. related burn group, ANOVA.

Inflammatory mediators play an important role in the pathogenesis of burn-induced remote ALI. The concentrations of TNF-α and IL-8 in BALF and serum were determined by ELISA. The TNF-α and IL-8 in BALF began to increase at 12 h after burn injury and maintained high concentrations up to 48 h post burn compared with those in sham burn controls. These concentrations were significantly reduced in the burned rats that received sodium butyrate (Figure 2A and 2B). In line with the measurements of BALF, the serum TNF-α and IL-8 concentrations of the burned animals were significantly higher than those of the sham animals 12 h to 48 h post injury. The increased TNF-α and IL-8 serum concentrations were markedly decreased by the sodium butyrate treatment (Figure 2C and 2D).

### Sodium Butyrate Protects against Histopathologic changes Induced by Severe Burn Injury

**Figure 3:**
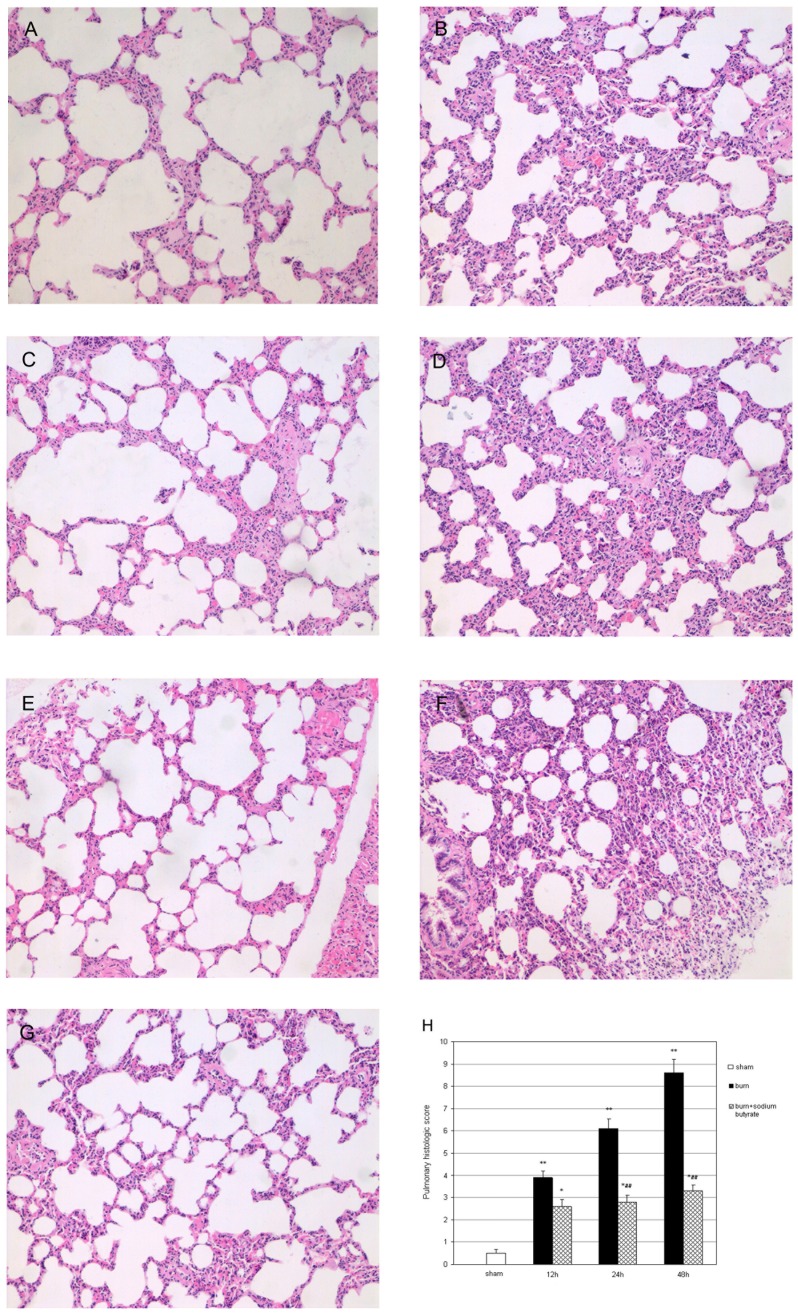
Effect of sodium butyrate on histopathologic changes in rats with burn-induced ALI (×100). Representative photomicrographs showing hematoxylin and eosin staining: lung edema, alveolar hemorrhage, and neutrophil infiltration. The epithelial and endothelial cell structure was destroyed in the burn group. Meanwhile, these changes were not identified or were less severe in the burn plus sodium butyrate group. The gradually increase in pulmonary histologic score started at 12 h after burn injury, and was significantly attenuated by sodium butyrate treatment. (A) Sham group, (B) burn group at 12 h post burn, (C) burn plus sodium butyrate group at 12 h post burn, (D) burn group at 24 h post burn, (E) burn plus sodium butyrate group at 24 h post burn, (F) burn group at 48 h post burn, (G) burn plus sodium butyrate group at 48 h post burn, and (H) changes in the pulmonary histologic scores. Results are given as mean ± SEM (n = 8). *p<0.05, **p<0.01, vs. sham, ANOVA; ^##^p<0.01, vs. related burn group, ANOVA.

No destructive changes were observed in the hematoxylin and eosin–stained lung tissues from the sham group (Figure 3A). As illustrated in Figure 3B, 3D, 3F, the lungs of the burned rats show marked proinflammatory alterations characterized by lung edema, alveolar hemorrhage and neutrophil infiltration, and destruction of the epithelial and endothelial cell structure. These histopathologic changes progressed gradually after burn injury. By contrast, a significant decrease was demonstrated in multiple features of ALI in sodium butyrate-treated rats (Figure 3C, 3E, and 3G). Neutrophil infiltration, interstitial edema, and pulmonary hemorrhage were all improved by sodium butyrate administration.

The pulmonary histologic scores of the burn group using the Gloor double blind score system gradually increased after thermal injury (Figure 3H). These increases were significantly attenuated by sodium butyrate administration. The pulmonary histologic scores of the burned rats that treated with sodium butyrate were significantly lower than those of related burn group at 24 and 48 h post burn.

### Sodium Butyrate Reduces Pulmonary Edema Induced by Severe Burn Injury

**Figure 4:**
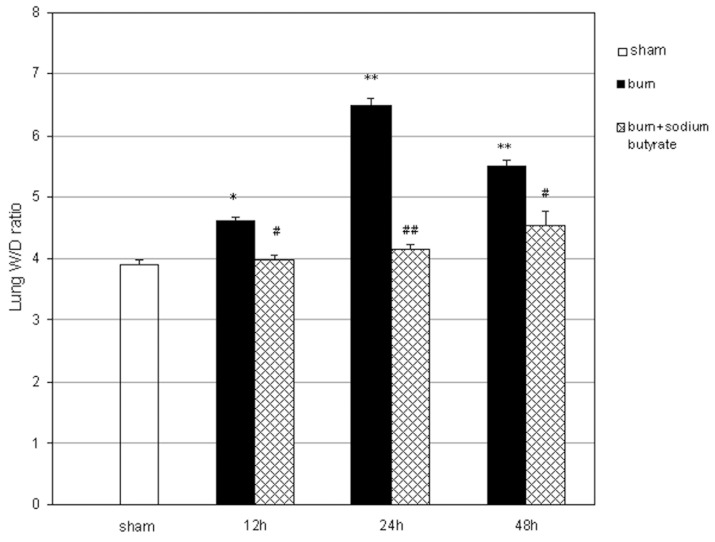
Effect of sodium butyrate on pulmonary edema after burn injury. The lung W/D ratios were calculated to evaluate lung edema. The 30% TBSA full-thickness burn resulted in a significant increase in lung W/D ratios, which were attenuated by the sodium butyrate treatment. Results are given as mean ± SEM (n = 8). *p<0.05, **p<0.01, vs. sham, ANOVA; ^#^p<0.05, ^##^p<0.01, vs. related burn group, ANOVA.

Pulmonary edema is one of the most characteristic pathologic changes in burn-induced remote ALI. The lung W/D ratio was examined to determine the effect of sodium butyrate on pulmonary edema. After a 30% TBSA full-thickness burn, the lung W/D ratio began to increase at 12 h after the injury, peaked at 24 h, and then decreased at 48 h (Figure 4). These increases were diminished in the burned rats treated with sodium butyrate. The lung W/D ratio was not significantly different between the burn plus sodium butyrate group and the sham group.

### Sodium Butyrate Inhibits HMGB1 Expression in the Lungs of Severely Burned Rats

**Figure 5:**
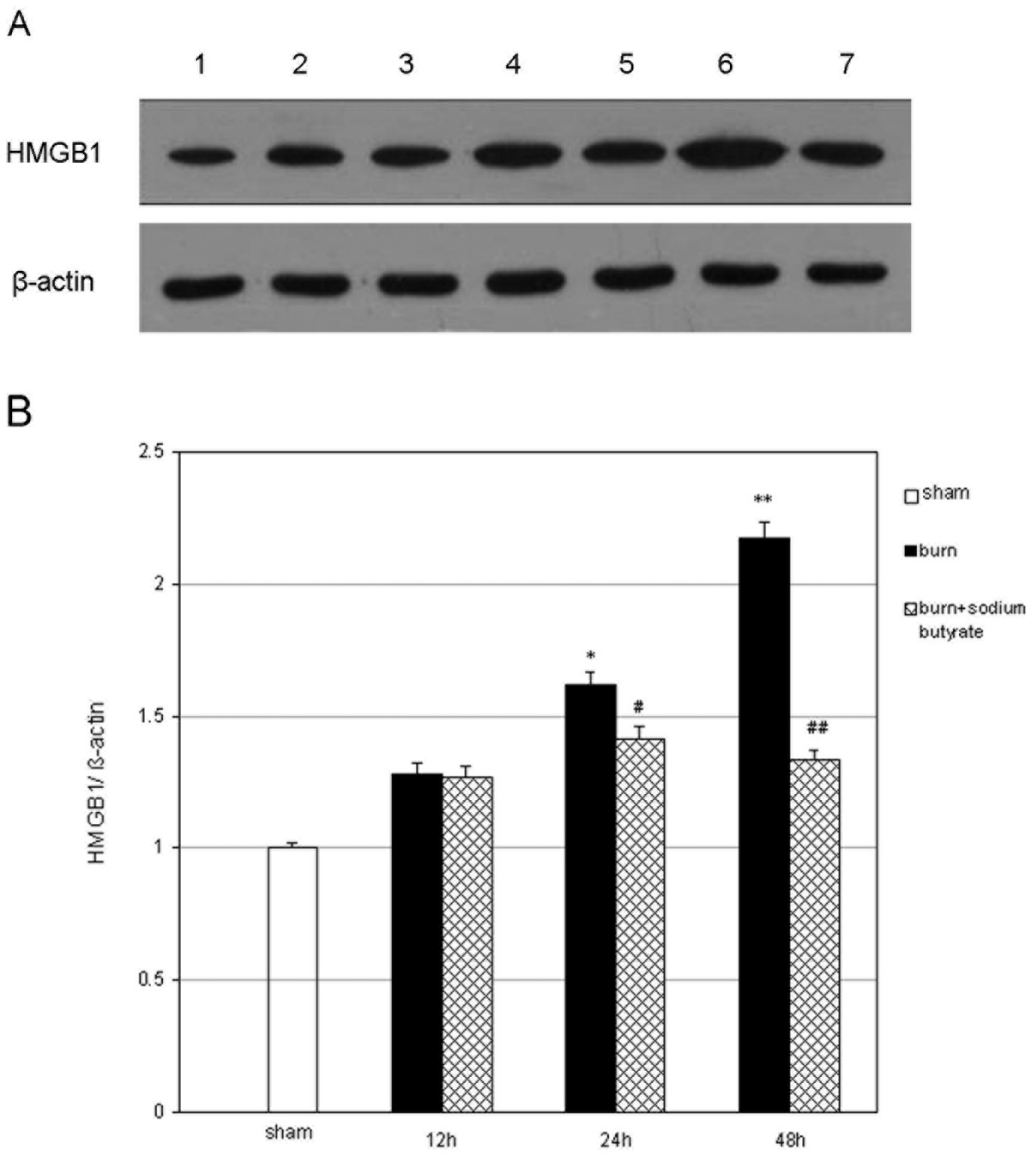
Effect of sodium butyrate on HMGB1 expression in lung tissues. HMGB1 expression in the lungs was evidently increased in the burn group compared with the sham group. Sodium butyrate treatment resulted in a marked decrease in pulmonary HMGB1 expression. (A) A representative results: Lane 1, sham group; Lane 2, burn group at 12 h post burn; Lane 3, burn plus sodium butyrate group at 12 h post burn; Lane 4, burn group at 24 h post burn; Lane 5, burn plus sodium butyrate group at 24 h post burn; Lane 6, burn group at 48 h post burn; Lane 7, burn plus sodium butyrate group at 48 h post burn. (B) Results from the independent experiments and were given as mean ± SEM (n = 8). *p<0.05, **p<0.01, vs. sham, ANOVA; ^#^p<0.05, ^##^p<0.01, vs. related burn group, ANOVA.

The β-actin expression in the lungs of the three groups seemed similar. After 30% TBSA full-thickness burn injury, HMGB1 expression was evidently increased in the burn group compared with the sham group, which was markedly decreased by sodium butyrate treatment (Figure 5A). Densitometric scan results show that the HMGB1/β-actin ratios in the burn group at 12, 24, and 48 h after injury were 1.28, 1.62, and 2.17 times those in the sham group, respectively. The HMGB1 protein expression levels were significantly lower in the sodium butyrate intervention group than those in the burn group at 24 and 48 h post burn (Figure 5B).

### Sodium Butyrate Reduces the Activities of MPO in the Lung of Severely Burned Rats

**Figure 6:**
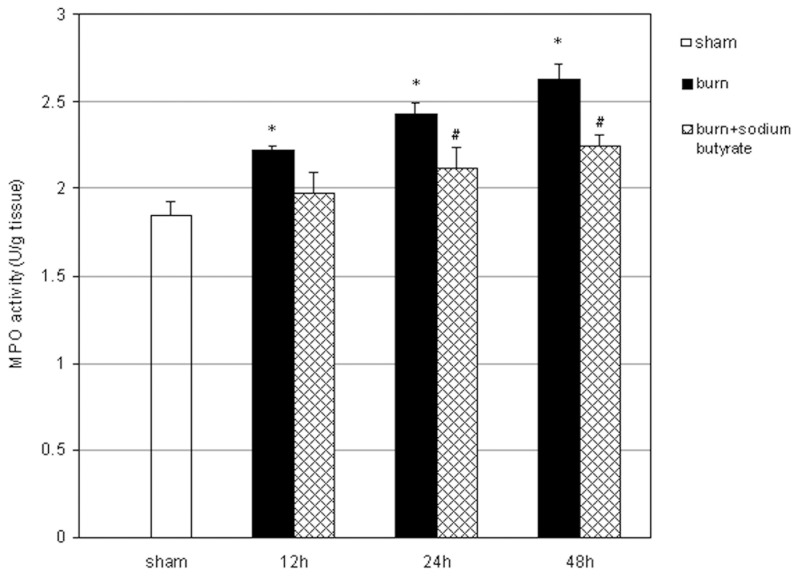
Effect of sodium butyrate on MPO activity in lung tissues. Pulmonary MPO activity started to increase gradually at 12 h after burn injury, which was markedly attenuated by sodium butyrate treatment. Results are given as mean ± SEM (n = 8). *p<0.05, vs. sham, ANOVA; ^#^p<0.05, vs. related burn group, ANOVA.

Neutrophil accumulation in the lungs was evaluated with MPO activity assays. Compared with the sham group, the activities of MPO in the lung began to increase markedly at 12 h after burn injury and maintained high concentrations up to 48 h post burn. These increases were evidently reduced by sodium butyrate treatment. The activities of MPO were significantly lower in sodium butyrate intervention group than those in burn group at 24 and 48 h post burn (Figure 6).

### Sodium Butyrate Attenuates the Pulmonary Oxidative Stress Induced by Severe Burn Injury

**Figure 7:**
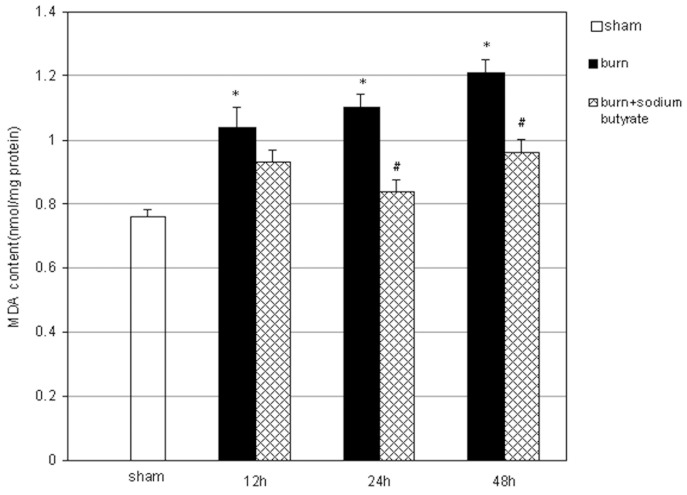
Effect of sodium butyrate on the MDA concentration in lung tissues. Pulmonary MDA concentration started to increase gradually at 12 h after burn injury, which was markedly attenuated by sodium butyrate treatment. Results are given as mean ±SEM (n = 8). *p<0.05, vs. sham, ANOVA; ^#^p<0.05, vs. related burn group, ANOVA.

The MDA concentration in the lung tissue was assessed to determine the effects of sodium butyrate on burn-induced pulmonary oxidative stress. Compared with the sham group, the pulmonary MDA concentration began to increase significantly at 12 h post burn and it remained high until 48 h after burn injury. The concentrations were evidently reduced by sodium butyrate treatment. The pulmonary MDA concentrations were significantly lower in burn plus sodium butyrate group than those in the burn group at 24 and 48 h post burn (Figure 7).

## Discussion

Burn injury is an important global healthcare problem. The lungs are frequently the first organs to fail after burns [27], and ALI always develops in major burn patients, even in the absence of inhalation injury and infection [28]. Moreover, impairment of pulmonary functions, such as inflammatory response and severe hypoxemia, damages other organs and tissues and, thus, contributes to the progressive development of multiple organ dysfunction syndrome [29]. Identifying early mediators of burn-induced ALI and their underlying molecular mechanisms are of critical importance but are not fully understood. The lung injury model in this study, which involves a 30% TBSA full-thickness burn and 6 h delayed resuscitation, results in a dramatic increase in pulmonary edema and histologic changes.

Sodium butyrate is a 4-carbon fatty acid salt that naturally occurs in animal fat [23]. This short-chain fatty acid butyrate inhibits cell growth and autophagy [30], [31], and induces cell differentiation and apoptosis in multiple cancer cell lines [32], [33]. Recently, sodium butyrate has been implicated in HMGB1 expression [20]–[23]. In the present study, we found that *in vivo* sodium butyrate administration inhibited pulmonary HMGB1 expression, decreased the lung W/D ratio, reduced the pulmonary histologic scores, and improved the pathologic changes.

HMGB1 is a nuclear factor that is extracellularly released as an early endogenous alarmin of inflammation following injury [34]. HMGB1, is released later than other proinflammatory cytokines, and serves as a “late mediator” of sepsis [12]. Blocking HMGB1 activity reduces mortality in an animal endotoxemia model, even when administered late during the course of the disorder [35]. Up to now, the emerging evidence suggests the pivotal role of HMGB1 in the development of the LPS-induced ALI [13]–[16]. In the present study, the expression of HMGB1 was evidently increased in the lung tissues concomitant with the increase in burn-induced lung injury. This result is consistent with that by Huang and coworkers [36], [37], who reported that the HMGB1 concentrations in the plasma and its mRNA expression in peripheral blood mononuclear cells were both significantly increased after extensive burn injury, which were associated with the development of sepsis and fatal outcomes among major burn patients.

The ALI induced by severe burn with delayed resuscitation is considered a secondary consequence of cutaneous thermal injury and is associated with acute systemic inflammatory response syndrome [38], [39]. Severe burn injury with delayed resuscitation triggers an excessive inflammatory response that manifests as increased concentrations of local and systemic inflammatory mediators [5]. In the current study, the TNF-α and IL-8 concentrations were significantly increased in the BALF and serum of rats with burn-induced ALI, which are associated with increased HMGB1 expression in the lung tissue. Treatment with sodium butyrate, an HMGB1 inhibitor, decreased the TNF-α and IL-8 concentrations both in the BALF and in the serum, and attenuated burn-mediated lung injury. The current results suggest that HMGB1 induced by thermal injury may be involved in regulation of the TNF-α and IL-8 productions in the lung, which further confirms that extracellular HMGB1 acts as a potent proinflammatory cytokine that contributes to the pathogenesis of ALI.

Excessive release of early inflammatory mediators triggers and intensifies the pulmonary inflammatory cascade during ALI [28]. The proinflammatory cytokine TNF-α induces the production of other inflammatory cytokines and stimulates the migration and adherence of neutrophils to endothelial cells [40]. IL-8, a proinflammatory CXC chemokine, is the main chemotactic factor and activator of neutrophils in the lung [41]. Consequently, activated neutrophils induce microvascular and pulmonary damage by secreting metalloproteinases, MPO, and collagenases, as well as ROS and nitrogen species during migration into alveolar spaces, thereby inducing endothelial permeability, which may lead to lung edema [41]. In the current study, pulmonary MPO activity was used to evaluate neutrophil infiltration into lung tissues. As a result, MPO activity was significantly increased after severe burn injury, which was abolished by the administration of sodium butyrate. These findings suggest that sodium butyrate lessen the infiltration of neutrophils into pulmonary tissues, which are attributed to the prevention of inflammatory mediators TNF-α and IL-8 productions.

In addition, burn injury and delayed resuscitation have been previously shown to cause excessive ROS generation and local burst of oxidation in the lungs, which are critical in pulmonary injury and associated with the development and progression of ARDS [6], [42]. Neutrophils are the major source of ROS production in ALI and ARDS [43]. ROS attack lipids, proteins, and DNA, causing irreversible oxidative stress in cells, which leads to lung injury [6]. Furthermore, ROS causes additional damage to the lungs by inducing the production of inflammatory cytokines through the activation of transcription factors such as nuclear factor-κB and activator protein-1 [43]. In the present study, the cellular injury caused by the release of ROS because of lipid peroxidation and oxidative stress was estimated in terms of the MDA concentrations in lung homogenates of rats subjected to burn injury and delayed resuscitation. We found that the MDA concentration in the lung tissues increased significantly after severe burn and delayed resuscitation, which was evidently reduced by sodium butyrate treatment. The present data, to our knowledge, is the first reveal that HMGB1 is involved in the oxidative stress of burn-induced remote ALI. However, in this study, we did not obtain enough data to elucidate whether the inhibition of oxidative stress by sodium butyrate is due to the reduction in inflammatory mediators caused by burn injury. This issue will be addressed in further studies.

In summary, sodium butyrate attenuates inflammatory responses, neutrophil infiltration, and oxidative stress in the lungs, and protects against remote ALI induced by severe burn, which is associated with inhibiting HMGB1 expression.
